# Supplementary material for: Carbapenem-resistant Escherichia coli exhibit diverse spatiotemporal epidemiological characteristics across the globe
Source: Commun Biol. 2024 Jan 6;7:51. doi: 10.1038/s42003-023-05745-7 (PMC10771496; doi:10.1038/s42003-023-05745-7)
Supplement: Supplementary file 2 — Supplementary Information [file 42003_2023_5745_MOESM2_ESM.pdf]

## Supplementary Figure Legends

**Supplementary Fig. 1 ST distribution on branches of the phylogenetic tree of CRECs.** The phylogenetic tree contains 268 representative CRECs from each branch of the evolutionary tree of the 17 dominant ST strains, spanning 3-5 continents.

**Supplementary Fig. 2 Dissemination patterns of ST38-CREC on four continents.** Intercontinental STs were classified as those with more than 10 CREC strains spreading across each continent. Countries indicated by red dots are those designated as having transmitted CRECs to five or more countries with a total of more than 10 strains.

**Supplementary Fig. 3 Dissemination patterns of ST10-CREC on three continents.** Intercontinental STs were classified as those with more than 10 CREC strains spreading across each continent.

Supplementary Fig. 1

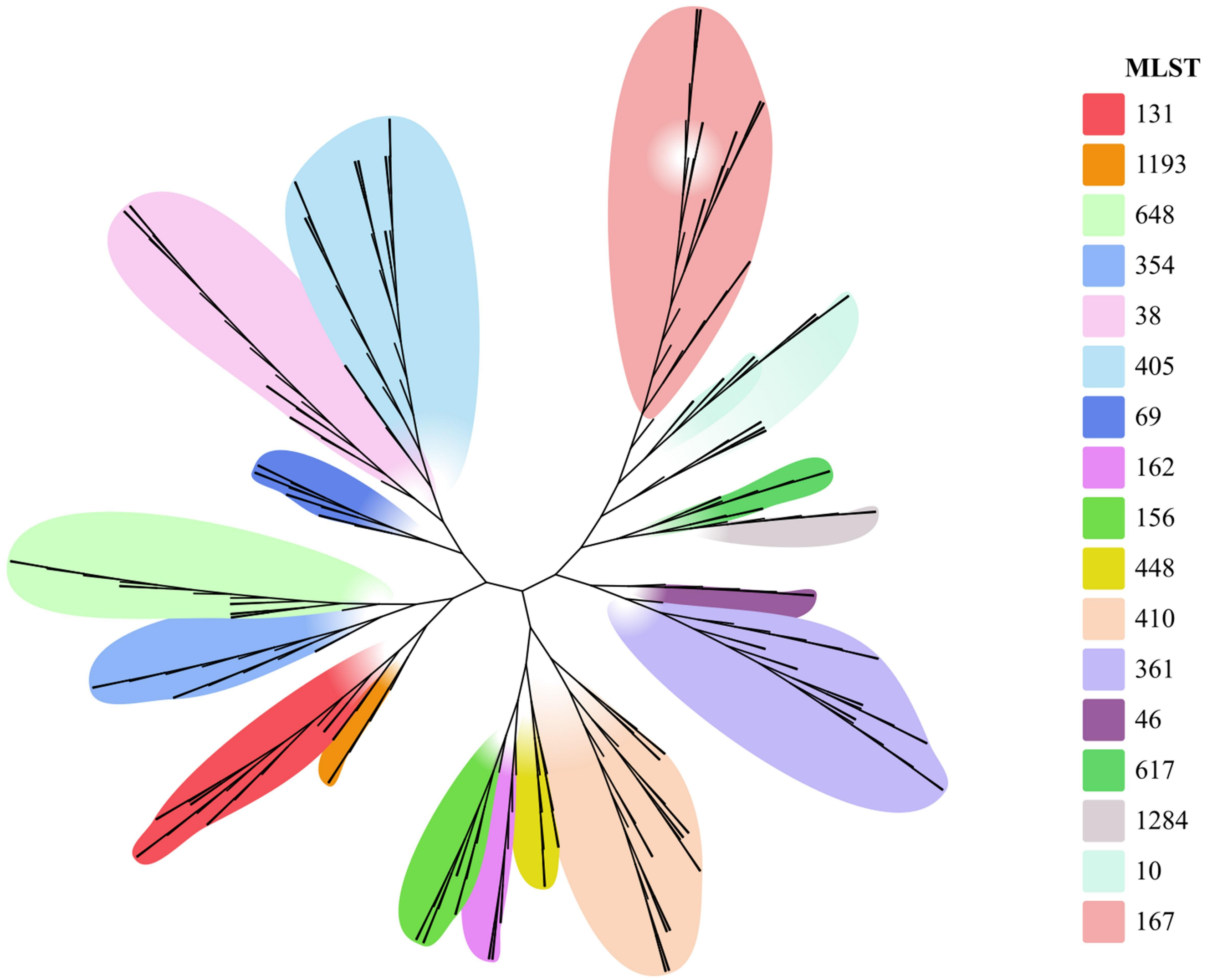

Supplementary Fig. 2

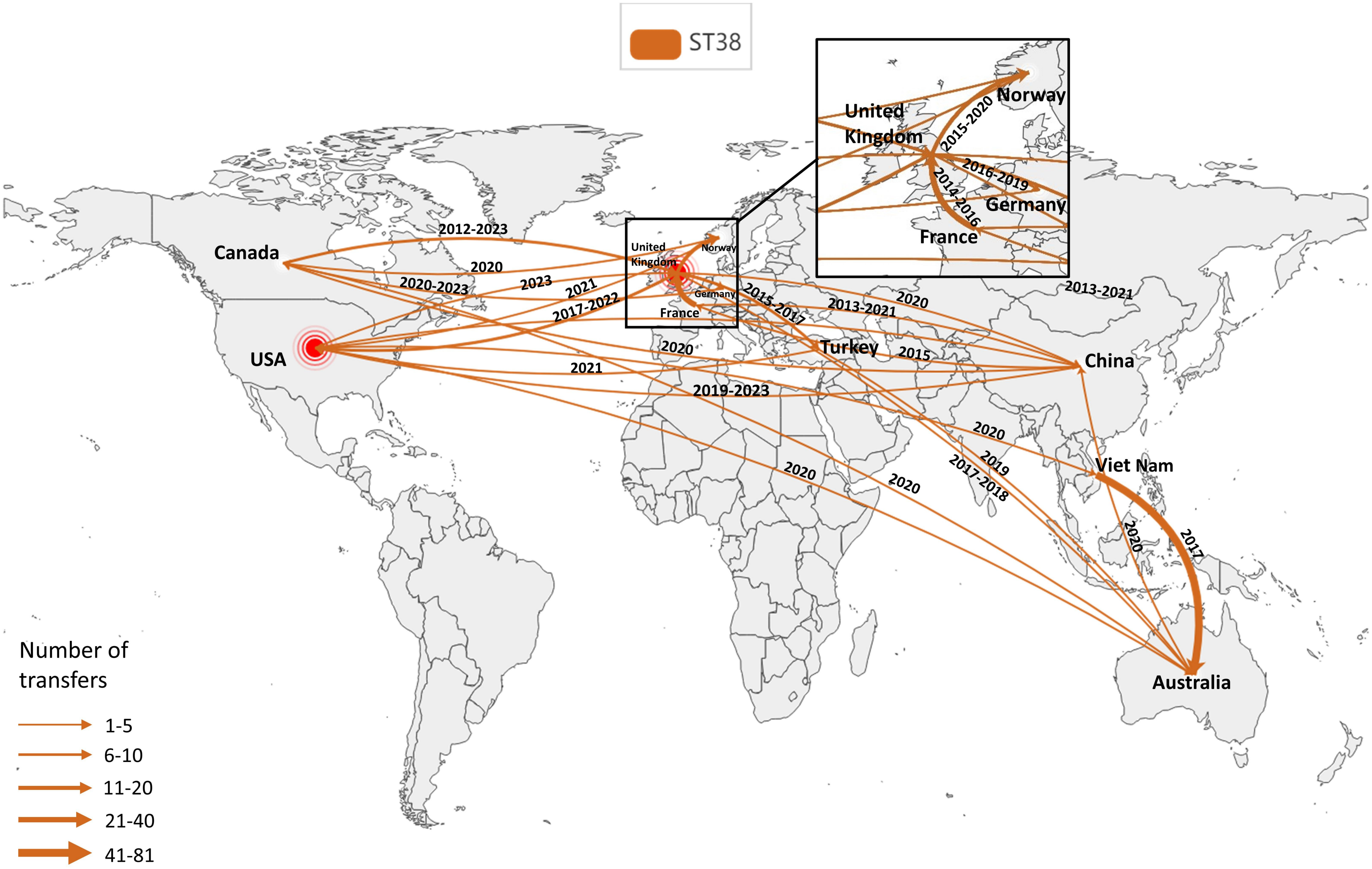

Supplementary Fig. 3

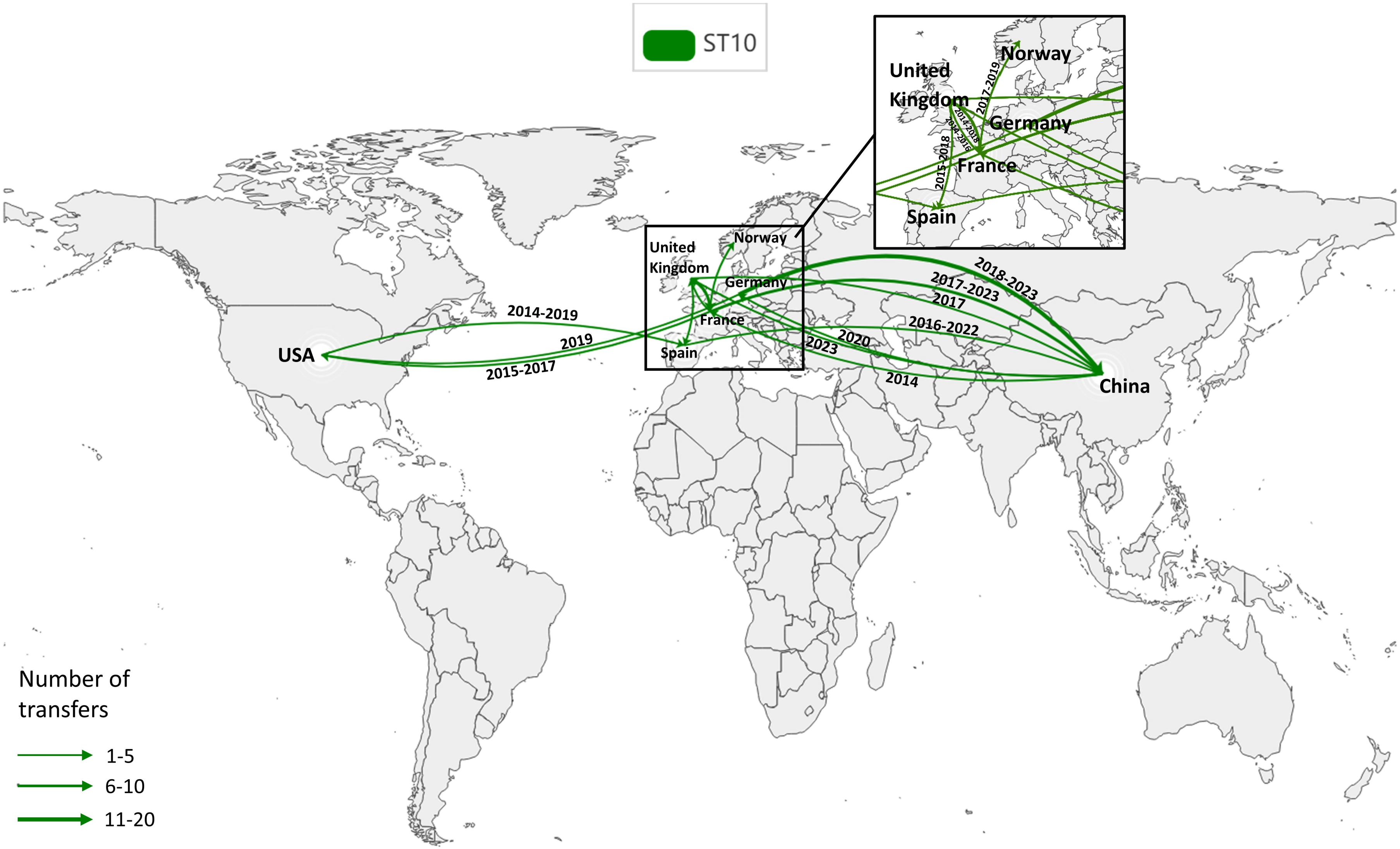

**Supplementary Note 1: Code and algorithm**

**Bactdating**

We utilized time-stamped phylogenetic trees and host ancestry reconstruction to analyze the temporal and locational data of CREC strains within a particular tree branch.

The specific commands parameters in R language are listed below:

First input the phylogenetic tree,which can be loaded from a Newick file using the commands from the ape package: t=read.tree('filename.nwk').Then calculate sum (t$edge.length) to determine if the branch length measurement is correct. Below 1 it needs to be adjusted using the command t$edge.length=t$edge.length*L,where L is the number of sites which were used to build this tree.Because this should be the total number of substitutions throughout the tree, so if we get a value below one or even only a bit above one, our branch lengths are probably not in the correct unit.

The second required input the dates at which the isolates were sampled. We used the decimal_date function of the lubridate package to convert the other date formats to decimal years. All strain names entered names(d) correspond to the isolation dates entered previously.

Then run BactDating using the main command:

Find an optimal root using: rooted=initRoot(t,d)

Resolving Multiple Groupings: rooted=multi2di(rooted)

Function Reference URL: https://rdrr.io/cran/ape/man/multi2di.html

Evaluating the Strength of the Time Signal Using Root-to-Tip Linear Regression:

r=roottotip(rooted,d)

Prediction with BactDating's MCMC algorithm:res=bactdate(rooted,d)

Check that the traces of the parameter look stable:plot(res,'treeCI')

**Speculative propagation trajectories of CRECs**

After constructing the time trees, we tracked the time and location information of CRECs on the same branch. Then we modeled the path of diffusion from the geographic locations of early-emerging strains to the geographic locations of late-emerging strains.

We customised the algorithm for obtaining propagation paths from evolutionary trees: mapping functions: f(treenodes) -> collection of (source country, target country, number, date range)

input: bactdating phylogeny tree

output: a collection of propagation path with source country, target country, number and date range (in years).

Algorithm:

Identify the similar genes on the same branch (if only one, search the parent branches), figure out the meta path of the gene with the earliest date as source country and the one with the latest date as the target country. Then merge the meta paths with the same source and target into one propagation path. The number of the propagation path is the summary of these meta paths. The date range of the propagation path is the from the earliest year of the meta path to the latest year of the meta path.

Notice: the meta paths with the same countries but the source and the target are reverse are considered as different propagation paths. In the graph they are displayed as two curves in the opposite direction.
