# Supplementary material for: Carbapenem-resistant Escherichia coli exhibit diverse spatiotemporal epidemiological characteristics across the globe
Source: Commun Biol. 2024 Jan 6;7:51. doi: 10.1038/s42003-023-05745-7 (PMC10771496; doi:10.1038/s42003-023-05745-7)
Supplement: Supplementary file 3 — Description of Additional Supplementary Files [file 42003_2023_5745_MOESM3_ESM.pdf]

Description of Additional Supplementary Files

File name: Supplementary Data 1
Description: The strain list and information used from the NCBI Pathogens database.

File name: Supplementary Data 2
Description: The source data behind Fig 1b in the paper.

File name: Supplementary Data 3
Description: The source data behind Fig 1c in the paper.

File name: Supplementary Data 4
Description: The source data behind Fig 1d in the paper.

File name: Supplementary Data 5
Description: The source data behind Fig 1e in the paper.

File name: Supplementary Data 6
Description: The source data behind Fig 2a in the paper.

File name: Supplementary Data 7
Description: The source data behind Fig 2a in the paper.

File name: Supplementary Data 8
Description: The source data behind Fig 3 in the paper.

F**ile name:** Supplementary Data 9
Description: The source data behind Fig 4 in the paper.
